# La place de la double arthrodèse dans la prise en charge du pied bot varus équin invétéré

**DOI:** 10.11604/pamj.2014.19.212.4508

**Published:** 2014-10-27

**Authors:** Karima Atarraf, Mounir Arroud, Lamiae Chater, My Abderrahmane Afifi

**Affiliations:** 1 Faculté de Médecine et de Pharmacie, Université Sidi Mohammed Ben Abdullah, Service d’ Orthopédie Pédiatrique, CHU Hassan II, Fès, Maroc

**Keywords:** Double arthrodèse, pied bot, varus, équin, invétéré, double arthrodesis, clubfoot, varus, equine, inveterate

## Abstract

La prise en charge du pied bot varus équin invétéré ( PBVEI) pose d’énormes problèmes thérapeutiques. La double arthrodèse sous-talienne et médio-tarsienne longtemps considérée comme la solution de sécurité pour ces déformations est encore couramment utilisée. Nous rapportons une série de 13 enfants opérés pour un pied bot varus équin invétérés (16 pieds) par Arthrodèse sous-talienne et médio tarsienne réalisée au service d'orthopédie pédiatrique du CHU Hassan II; de Fès au Maroc sur une période de 4 ans; étalée de janvier 2009 à décembre 2012. L’âge moyen de nos patients était de 12,6 ans avec prédominance féminine. L'origine congénitale était retrouvée chez 10 patients. L'atteinte était gauche chez 8 patients avec une localisation bilatérale chez 3 patients. La radiographie standard du pied de face et de profil a révélée une divergence talo-calcanéenne qui variait entre 5 et 20°, l'angle talus-1er métatarsien entre 20 et 40° (avec une moyenne de 28°) et l'angle calcanéus-5ème métatarsien entre 15° et 45° (avec une moyenne de 30°). Tous les patients ont bénéficiés d'une arthrodèse sous-talienne et médio tarsienne. Les résultats étaient satisfaisants dans 98% des cas. Le pied était plantigrade dans 9 cas, le varus de l'arrière pied persistait dans 4 pieds alors que l’équin et le varus de l'avant pied étaient notés chez 2 cas. La double arthrodèse est l'intervention idéale pour stabiliser et corriger les déformations rencontrées dans le PBVE invétéré, elle assure totalement le verrouillage du couple de torsion. Elle permet outre une correction des diverses déformations et une ré-axation de l'arrière-pied dans les 3 plans de l'espace.

## Introduction

L'arthrodèse sous-talienne et médio tarsienne est indiquée dans le traitement du pied bot varus équin invétéré comme une intervention de sauvetage permettant une correction des déformations et procurant ainsi des résultats meilleurs qu'en cas de réalisation d'une libération des parties molles ou des ostéotomies. Et sous le terme de pied bot varus équin invétéré (PBVEI), on désigne un pied bot diagnostiqué après la marche. on rattache à cette entité, les déformations résiduelles d'un pied bot précédemment traité (hypo correction ou hypercorrection qu'elle qu'en soit la modalité d'expression) ou encore la récidive complète après traitement.

## Méthodes

Nous avons réalisé une étude rétrospective portant sur 13 malades (16 pieds) suivis au service d’ Orthopédie Pédiatrique du CHU Hassan II; de Fès pour pied bot varus équin invétéré sur une période de 4 ans (janvier 2009-décembre 2012) opérés par double arthrodèse sous-talienne et médio-tarsienne.

## Résultats

L’âge moyen de nos patients était de 12,6 ans avec des extrêmes entre 10 et 15 ans. 7 malades étaient de sexe féminin, soit 58.3%. Deux de nos patients avait une notion de souffrance néonatale dont l’évolution était marquée par un retard psychomoteur et l'apparition d'une déformation progressive des pieds. Alors qu'un autre avait présenté un accident vasculaire cérébral ischémique sylvien gauche ayant gardé une hémiplégie droite avec déformation progressive du pied en varus équin.

**Figure 1:**
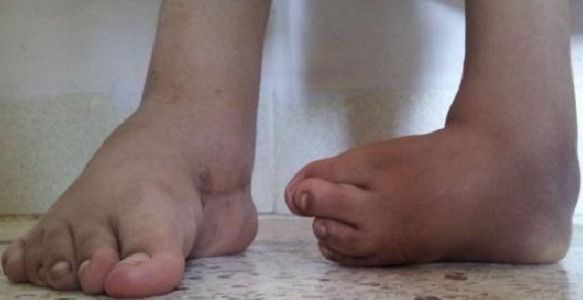
Fille de 11 ans, ayant un Pied bot varus équin bilatérale opérée à droite avec bon résultat et revient pour le pied gauche

**Figure 2:**
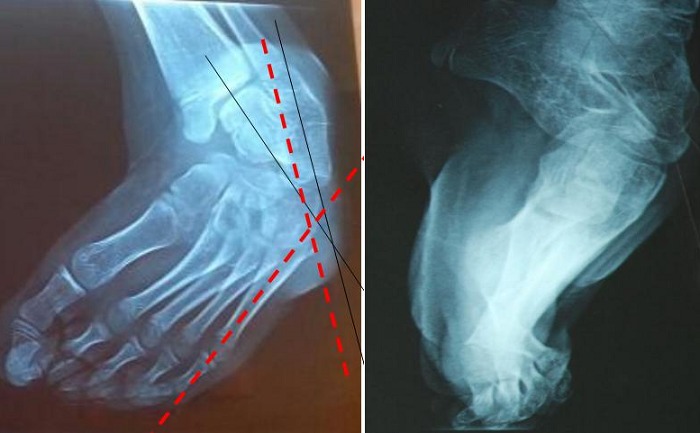
Aspect radiologique du pied gauche

Le diagnostic était fait à la naissance chez 12 patients, alors qu'il n’était fait qu’à l’âge de 8 ans chez un patient. La bilatéralité a été notée chez 3 patients (Figure 1). L’équin variait entre 45° et 90°; Le Varus de l'arrière pied variait entre 35° et 90° et la rotation du bloc calcanéo-pédieux était entre 45 et 90° alors que L'adduction de l'avant pied variait entre 20 et 45 °. Sur le plan radiologique; La divergence talo-calcanéenne variait entre 5° et 20°, L'angle talus-1er métatarsien entre 20° et 40° et l'angle calcanéus-5ème métatarsien était entre 15 et 45° (Figure 2).

**Figure 3:**
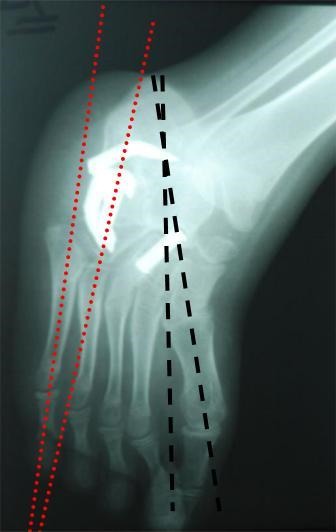
Radiographie après double arthrodèse et ostéosynthèse par 4 agrafes de blount angle talus-1er métatarsien à 5° et angle calcanéus-5éme métatarsien à 15

Les 10 patients (77%) qui avaient un pied bot varus équin idiopathique avaient bénéficié à leur jeune âge d'un traitement orthopédique puis chirurgical par libération des parties molles, alors que les 3 autres patients (23%) n'avait bénéficié d'aucun traitement. La voie d'abord antérolatérale a été utilisée dans toutes les intervention Permettant d'avoir toutes les structures nerveuses et tendineuse sous contrôle; et permettant de rétablir la divergence talo-calcanéenne dans les trois plans et de corriger le varus existant entre le talus et le calcanéus. une foie La correction de l'avant pied est obtenue; elle est alors fixée par trois agrafes (Figure 3). Une agrafe calcanéo-cuboidienne latérale mise bien horizontalement et une agrafe dans la tête du talus et dans le corps de l'os naviculaire, mise obliquement de dehors en dedans et une troisième dans l'articulation sous talienne. La consolidation était obtenue en 2 mois.

**Figure 4:**
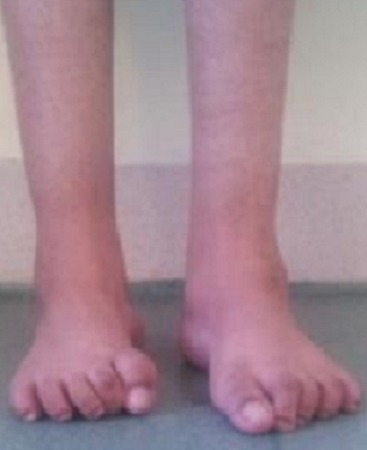
Aspect final avec deux pieds plantigrades

L'infection de la plaie était notée dans 6 cas et était traitée par soins locaux et une antibiothérapie adaptée à l'antibiogramme qui était positif à staphylococcus aureus; nous n'avons rencontré aucun cas d'infection ayant évolué jusqu’à l'ostéite. Par contre chez deux malades nous avons noté une nécrose cutanée, et le traitement consistait en une nécrosectomie puis cicatrisation dirigée. Aucune complication post opératoire tardive n'a été constatée (pas de nécrose du talus ni de raideur de la cheville). L'apparition de la douleur après une marche longue était présente uniquement chez 3 patients dont un a bénéficié d'une ablation des agrafes. Le varus de l'arrière pied persistait dans 4 pieds (25%) le varus de l'avant pied persistait dans 1 pied (6%).l’équin persistait dans 2 pieds (13%). Le résultat était jugé très bon pour 6 pieds (34%); bon pour 9 pieds (58%) (Figure 4) et mauvais dans un seul cas (8%).

## Discussion

L'arthrodèse sous-talienne et médio tarsienne est indiquée dans le traitement du PBVEI comme un moyen de sauvetage permettant une correction des déformations et procurant ainsi des résultats meilleurs. Cette technique n'est réalisée qu'en fin de maturation entre l’âge de 10 et 12 ans afin d’éviter toute perturbation de la croissance. Ce qui est le cas dans notre série où l’âge moyen des patients était de 12,6 ans rejoignant la série de HERSH [1] où l’âge des patients au moment de l'intervention variait entre 10 et 16 ans, et dans la série de RIBAULT,L [2] où l'arthrodèse était réalisée chez 2 patients dont l’âge était de 12 et 14 ans, Contrairement à la série de BITARIHO [3] faite à UGANDA où elle était réalisée à un bas âge.

Dans la collection nouveau traité de technique chirurgicale dirigée par MERLE D'AUBIGNE et datant de 1976, SERINGE R [4] à propos de la double arthrodèse tardive pour pied bot a écrit ceci « la réalisation correcte d'une double arthrodèse ne saurait se faire sans utiliser deux voies d'abord distinctes: l'une externe habituelle à toute double arthrodèse, l'autre interne permettant une libération interne et un allongement du jambier postérieur ». Dans notre série la voie d'abord consistait en une incision externe à 1cm au dessous de la malléole externe et étendue en avant à la face dorsale du pied à la partie la plus antérieure de l'articulation talo-naviculaire; une ostéotomie talo-naviculaire et calcanéo -cuboidienne est réalisée puis une fixation de la correction obtenue est réalisée par la mise en place de 3 agrafes de BLOUNT par la suite une fermeture des différents plans est réaliser sur un drain de Redon après avoir assurer l'hémostase. Cette même technique était réalisée chez tous les patients de la série de HERSH [1]. Une attelle plâtrée est réalisée en fin d'intervention, le contrôle radiologique était fait dès que possible avec plâtre après l'acte opératoire pour vérifier la réduction et la position du matériel d'ostéosynthèse même chose a été rapportée dans la série de HERSH [1] où le membre était surélevé pendant 3 jours, suivi d'un contrôle radiologique.

L'infection de la plaie; due à l'infection de l'hématome vu que le drainage était insuffisant; était notée dans 6 cas traitée par soins locaux et une antibiothérapie adaptée à l'antibiogramme qui était positif à « staphylococus aureus »; nous n'avons rencontré aucun cas d'infection ayant évolué jusqu’à l'ostéite. Dans la série de HERSH [1] 2 cas d'infection de la plaie étaient notés dus dans un cas à « klebsiella » et dans le 2ème cas à « staphylococus » aureus traité par antibiothérapie adaptée à l'antibiogramme avec bonne évolution alors que REZZOUK [5] a signalé, dans son travail portant sur la correction des déformations sévères du pied par l'appareil d’ « ILIZAROV », 2 cas d'ostéite du calcanéum et 4 cas d'infection le long des broches. Trois cas de nécrose cutanée ont été notés dans notre série le long de la plaie traitée par cicatrisation dirigée expliquée par l’étendue de la dissection et surtout à la fermeture cutanée forcée. Cette complication est décrite par la plupart des auteurs qui ont travaillé sur ce sujet; même au cours de la correction par fixateur externe. C'est ainsi que, RIBAULT L [2] déplore 1 cas de nécrose cutanée au cours du traitement chirurgical, alors que HERSH déplore 14 cas qui ont été traités par soins locaux sans antibiothérapie.

Après cicatrisation complète un plâtre circulaire cruro-pédieux est confectionné au 10ème jour, alors que dans la série de HERSH [1] elle a été réalisée entre le 10ème et le 14ème jour. Un contrôle radiologique a été fait à la 6ème semaine puis une immobilisation a été réalisée pour le reste de la durée qui a été de 8 semaines; une période au cours de laquelle l'appui était autorisé alors qu'elle était de 12 semaines pour HERSH [1] et parfois plus jusqu’à création de l'arthrodèse. Aucun cas de nécrose du talus; comme complication fréquente et non négligeable de la double arthrodèse; n'a été noté dans notre série, de même que pour la pseudarthrose; ceci pourrait s'expliquer par deux attitudes essentielles la résection osseuse importante imposée par l'importance des déformations; et la stabilisation efficace du montage par agrafe de BLOUNT et botte plâtrée pendant deux mois. Sur le plan morphologique une correction plantigrade a été obtenue dans 9 interventions suites auxquelles les patients ont repris une fonction normale par la suite. Alors que dans la série de HERSH [1] 75 pieds sur 80 ont repris leur activité complète. La persistance de l’équin était notée chez deux patients (qui était bien toléré) il s'agit d’ une conséquence habituelle de l'arthrodèse sous talienne et médio-tarsienne. Le varus résiduel de l'arrière pied peut être imputable à des erreurs d'appréciation au moment des la résection osseuse et de la contention. Ces erreurs résultent des difficultés de réglage puisque l'opération est exécutée sur un patient couché. Il faut tenir compte aussi du fait que dans tous les pieds bot varus équins invétérés sévères, il n'y a pas que des anomalies de la position des os du tarse mais des déformations osseuses plus a moins sévères associées.

Dans notre série le varus de l'arrière pied persistait dans 4 pieds chose qui est due souvent à une déformation du calcanéum. L'ostéotomie décrite par DWYER qui nous est connu à travers le travail de SERINGE et coll semble être la seule solution pour la corriger [4] alors que Le varus de l'avant pied persistait dans un cas. La petite taille de notre échantillon et le faible nombre de résultats non satisfaisants ne nous a pas permis de trouver une relation entre le résultat et les paramètres comme l’âge, le sexe, l’étiologie. Au plus grand recul, les résultats sont restés satisfaisants. Ceci démontre qu'il s'agit d'une technique fiable dont le résultat satisfaisant se maintient dans le temps. nos résultats étaient très bon chez 4 patients, bon chez 8 patients, mauvais chez 1 patient qui gardait une douleur limitant le périmètre de marche. ces résultats se rapprochent de ceux obtenus par la correction à l'aide du fixateur externe d'ILIZAROV. C'est ainsi que LA VILLE JM [6] a noté un résultat satisfaisant dans 8 cas sur un total de 9 pieds varus équin de l'adulte et de l'adolescent. Et que PREVOT [7] a obtenu aussi avec le fixateur externe 20 cas de résultats satisfaisants sur un total de 21 PBVE.

## Conclusion

La double arthrodèse sous-talienne et médio-tarsienne est l'intervention idéale pour stabiliser et corriger les déformations de l'arrière-pied, elle assure totalement le verrouillage du couple de torsion. Elle permet en outre une correction des diverses déformations et une ré-axation de l'arrière-pied dans les 3 plans de l'espace.
